# Non-Linear Relationship between Economic Growth and CO_2_ Emissions in China: An Empirical Study Based on Panel Smooth Transition Regression Models

**DOI:** 10.3390/ijerph14121568

**Published:** 2017-12-13

**Authors:** Zheng-Xin Wang, Peng Hao, Pei-Yi Yao

**Affiliations:** 1 School of Economics, Zhejiang University of Finance & Economics, Hangzhou 310018, China; zxwang@zufe.edu.cn (Z.-X.W.); haoopeng@163.com (P.H.); 2 Center for Research of Regulation & Policy, Zhejiang University of Finance & Economics, Hangzhou 310018, China

**Keywords:** CO_2_ emissions, economic growth, non-linear relationship, PSTR model

## Abstract

The non-linear relationship between provincial economic growth and carbon emissions is investigated by using panel smooth transition regression (PSTR) models. The research indicates that, on the condition of separately taking Gross Domestic Product *per capita* (GDPpc), energy structure (Es), and urbanisation level (Ul) as transition variables, three models all reject the null hypothesis of a linear relationship, i.e., a non-linear relationship exists. The results show that the three models all contain only one transition function but different numbers of location parameters. The model taking GDPpc as the transition variable has two location parameters, while the other two models separately considering Es and Ul as the transition variables both contain one location parameter. The three models applied in the study all favourably describe the non-linear relationship between economic growth and CO_2_ emissions in China. It also can be seen that the conversion rate of the influence of Ul on *per capita* CO_2_ emissions is significantly higher than those of GDPpc and Es on *per capita* CO_2_ emissions.

## 1. Introduction

Since the implementation of the reform and opening-up policy, the Chinese economy has developed rapidly and China has become the largest developing country and the second biggest economic entity in the world. However, excessive consumption of energy and mass emission of pollutants are causing sustained environmental pollution and eco-deterioration in China as a result of this growth. China thus bears huge responsibilities for environmental protection during such economic development. In January, 2016, China Meteorological Administration released *China Greenhouse Gas Bulletin in 2014* in which it points out that the Chinese carbon emissions account for 28% of all global emissions, surpassing the sum of those from the USA (14%), and the European Union (12%). In response the Chinese Government has formulated a series of policies and goals. For example, the *Sino-U.S. Joint Declaration on Climate Change* proposed that CO_2_ emissions per unit GDP in 2030 are expected to decrease by 60% to 65% compared with those in 2005. In the long-term, for research into economic growth and environmental pollution, scholars generally establish models by using environmental Kuznets curve (EKC) theory while empirical result tends to show large disparities therewith. In this study, the panel smooth transition regression (PSTR) model proposed by González et al. [1] is applied to investigate the relationship between economic growth and carbon emissions in China. And the innovations of this study is using this model to examine the non-linear relationship under different transition variables. The PSTR model is more flexible than classical panel models and is good at describing cross-section heterogeneity. Furthermore, whether a non-linear relationship exists between the two factors is verified and the transition rates on the condition of taking various variables as thresholds are investigated. The rest of study is organised as follows: Section 2 contains a literature review, Section 3 summarises the models used and the data therefrom, Section 4 summarises the empirical results, and Section 5 draws the conclusions.

## 2. Literature Review

Among the research into environmental problems and economic development, the EKC hypothesis is seminal: it proposes an inverted U-shaped curve mapping the relationship between environmental pollution and economic development. After referring to Kuznets’ work [2] on relationship between economic growth and income inequality, Grossman and Krueger [3] introduced the hypothesis into the analysis of environmental problems in 1991. Grossman and Krueger proposed a theory that changes in *per capita* income bring about two types of effects to explain the phenomenon. They suggested that economic development brings large-scale economic activities and resource demands, resulting in a negative effect on environmental quality. However, technologies develop and the economic structures correspondingly improve with growth. Therefore, an inverted U-shaped curve occurs due to the superposition of the two effects and among the empirical research on pollutant emission and economic development, numerous papers have covered the theory thereof. For example, the result of using inverted U-curves was obtained by multiple scholars including Grossman and Krueger [4], Perman and Stern [5], Stern [6], Galeotti et al. [7], and Yavuz [8]. However, other scholars acquired the results of an N-shaped mode of EKC such as Moomaw and Unruh [9], Martínez-Zarzoso and Bengochea-Morancho [10] while some scholars obtained an inverted N, such as Ajimi et al. [11].

Numerous scholars have criticised the inconsistent results among the various EKC curves, for example, Yang et al. [12] indicated that undersized data sets probably caused the relationship described by EKC hypotheses to be unsuitable for the construction of specific models. Furthermore, different models can be applied to different countries and regions in the world by constructing symbolic regressions on the data. Li et al. [13] and Apergis [14] noticed that heterogeneity is easily found in panel data from different countries and regions and they solved the problem by using traditional unit root and co-integration tests. However, González et al. [1] pointed out that it is invalid, or incomplete, to deal with the heterogeneity problem using these methods and furthermore, they proposed a new non-linear model (the PSTR model) to deal with the heterogeneity problem.

There are various publications about the relationship between economic growth and carbon emissions. Fernández-Amador et al. [15] investigated the relationship between GDP per capita and CO_2_ emission per capita associated with production and consumption activities. They found that carbon efficiency can gain from economic development. Ahamd [16] used cointegration analysis and vector error correction model to investigate the relationships among carbon emission, energy consumption and economic growth in India and found that a long run cointegration relationship exist. Ito [17] examined the linkage between CO_2_ emissions, economic growth and relevant variables by a dynamic panel model. Narayan et al. [18] used a cross-correlation estimates method to investigate the dynamic relationship between economic growth and carbon emissions for 181 countries. Zhu et al. [19] investigated the impact of foreign direct investment, economic growth and carbon emissions in five ASEAN countries by a panel quantile regression model. In this paper, different from the above studies, we consider the possible non-linear relationship between CO_2_ emissions and economic growth, and using three different PSTR models to examine it.

Changing with time and sample cross-section, the regression coefficients of PSTR models are heterogeneous and can be considered as the generalisation of panel threshold regression (PTR) models. For a PTR model, it assumes that there are obvious grouping characteristics among observed data, together with clear limits between different groups, and the threshold variables can be used for corresponding grouping [20]. Whereas, changes in the actual economy and other variables cannot strictly satisfy the hypothesis of the model, so regression results tend to be jumpy and discontinuous while using a PTR model to deal with practical problems, which does not conform to any practical law. However, the problem can be overcome by using the PSTR model. For the PSTR model, the changes in groups of variables not only reflect the jump in different regimes at the threshold but a transition function of threshold variables, which enable panel data to achieve smooth regime transition within a certain range. Therefore, the PSTR model is regarded as the generalisation of a PTR model and favourably conforms to economic realities. In addition, the PSTR model has obvious advantages over standard linear regression models for solving cross-section heterogeneity and time-instability problems. Heidari et al. [21] found that the relationship between CO_2_ emissions and economic growth conforms to the EKC hypothesis after fitting the relationship among economic growth, CO_2_ emissions, and energy consumption of five countries in the ASEAN bloc by using the PSTR model. Chiu [22] carried out PSTR modelling for the relationship between net incomes, energy use, and CO_2_ emissions by using panel data from 99 countries. Also, different countries are grouped and the EKC hypothesis verified between different groups.

## 3. Model and Data

### 3.1. Model Set-Up

The non-linear relationship between carbon emissions in China, and economic growth, is described by using the PSTR model. The fundamental PSTR mode containing two regimes is defined as follows:

(1) $$y_{it}=\mu_{i}+{\beta^{\prime }}_{0}x_{it}+{\beta^{\prime }}_{1}x_{it}G(q_{it};\gamma ,c)+\varepsilon_{it}$$

In Equation (1), *i* = 1,2,…,*N* and *t* = 1,2,…,*T* in which *N* and *T* refer to the total individual number and period number, respectively. *G* (*q_it_*; *γ, c*) represents a transition function, which is a continuous function with values between 0 and 1, where, *q_it_* is a transition variable. Additionally, *x_it_*, *μ_i_*, and *ε_it_* represent a *k*-dimensional exogenous variable, a fixed effect among individuals, and an error term, respectively.

The common functional forms of *G* (*q_it_*; *γ, c*) include exponential and logistic functions. In the study, a logistic function was applied with reference to Granger and Teräsvirta [23] and González et al. [1]:

(2) $$G\left(q_{it};\gamma ,c\right)={\left(1+\exp\left(-\gamma \prod_{j=1}^{m}\left(q_{it}-c_{j}\right)\right)\right)}^{-1}$$

where, *c* = (*c*_1_,…,*c_m_*)*^t^* refers to an *m*-dimensional location parameter vector and the slope parameter *γ* determines the smoothness of the transition function, namely, the transition rate from one regime to another. To identify the model, parameters *c* and *γ* are restricted, namely, *c*_1_ ≤ *c*_2_ ≤,…*≤ c_m_*, *γ >* 0. When the location parameter *c* is a fixed value, the estimation parameter of *x_it_* is smoothly transferred within the range of *β*_0_ ~ *β*_0_ + *β*_1_.

In empirical research, González et al. [1] suggested taking *m* = 1 or *m* = 2. At *m* = 1, *γ* → ∞, the transition function *G* (*q_it_*; *γ, c*) degenerates into an indicative function and has the value 1 for *q_it_* > *c_j_* and 0 otherwise. At *m* = 2, the transition function is symmetric with respect to the point (*c*_1_ + *c*_2_)/2 and has a minimum at that point while the function *G* (*q_it_*; *γ, c*) has a value of 1 when the transition variable *q_it_* has either large, or low, values. If *γ* → ∞, the model is transformed to a three-regime transition model and the two external regimes are the same, and differ from the middle regime. Generally, at *m* > 1, *γ* → ∞, there are two different regimes while the transition function *G* (*q_it_*; *γ, c*) has values in the range 0 to 1, and is 0 or 1 only at *c*_1_,…,*c_m_*. Additionally, under *γ* → 0, the transition function *G* (*q_it_*; *γ, c*) is a constant whatever the values of *m*, and model (1) degenerates into a panel fixed effect model. The PSTR model can be extended to a multi-regime model, shown as follows:

(3) $$y_{it}=\mu_{i}+{\beta^{\prime }}_{0}x_{it}+\sum_{j=1}^{r}{\beta^{\prime }}_{j}x_{it}G\left(q_{it}^{\left(j\right)};\gamma_{j},c_{j}\right)+\varepsilon_{it}$$

where, the logistic function form (2) is applied as the transition function $G\;\left(q_{it}^{\left(j\right)};\gamma_{j},c_{j}\right)$ with *j* = 1,…,*r*. When *m* = 1 and all slope parameters *γ_j_* → ∞, the model (3) degenerates to the PTR model proposed by Hansen [20]. Model (3) can be used to test residual heterogeneity.

Previous research into environmental problems and economic growth did not consider the possible missing variable problem. Thus, guided by the practice of Wu et al. [24], apart from traditional GDP *per capita* (GDPpc) and carbon emissions *per capita* (Rce), multiple variables are introduced including energy structure (Es), urbanisation level (Ul), trade openness (To), and marketisation degree or marketisation index (Mi). Here, GDPpc and Rce represent economic development level and pollution level, respectively.

In the study, the transition variable *q_it_* varies with changes of individuals and time, which allows the random change of regression parameters of different individuals in different periods. It is reasonable to measure the non-linear relationship between carbon emissions and economic growth in different provinces of China in different periods. Therefore, the equation is constructed as follows:

(4) $$\begin{array}{l} lnRce_{it}=\mu_{i}+\beta_{1}\ln RGDP_{it}+\beta_{2}\ln Es_{it}+\beta_{3}\ln Ul_{it}+\beta_{4}\ln To_{it}+\beta_{5}\ln Mi_{it}+\\ \left(\beta_{1}^{\left(1\right)}\ln RGDP_{it}+\beta_{2}^{\left(1\right)}\ln Es_{it}+\beta_{3}^{\left(1\right)}\ln Ul_{it}+\beta_{4}^{\left(1\right)}\ln To_{it}+\beta_{5}^{\left(1\right)}\ln Mi_{it}\right)\\ {}*G\left(q_{it}^{\left(1\right)};\gamma^{\left(1\right)},c^{\left(1\right)}\right)+\varepsilon_{it} \end{array}$$

The three models are established by taking logarithms of all variables in (4) and separately valuing the transition variable as ln*GDPpc*, ln*Es* and ln*Ul*. In the literature review, we known that CO_2_ emissions has strong relationship with economic growth. And before model construction, we have done the grey correlation analysis on all series, and found that among all the variables, as ln*GDPpc*, ln*Es* and ln*Ul* are highly correlated with ln*Rce*. Based on that, we choose as ln*GDPpc*, ln*Es* and ln*Ul* as the transition variables, respectively.

### 3.2. Data Specification 

The data were taken from the *China Statistical Yearbook*, *China Energy Yearbook*, and local statistical yearbooks from each province and region (except for Tibet) for the years from 1995 to 2015, inclusive. The specific descriptions of various variables are shown as follows:

(1) GDP *per capita* (GDPpc): the EKC curve reflects that the development of economic levels probably pollutes the environment while, with constant economic growth, the pollution levels increase at first and then decrease after the economic levels reach a turning point. The GDPpc is taken as the variable representing the economic growth levels. Based on 1995 constant prices, the practical GDP converted by GDP index *per capita* represents the income level in unit of yuan. Province-level GDP *per capita* can be found in *China Statistical Yearbook*.
(2) Energy structure (Es): restricted by natural resource endowment during energy production, coal is always the primary constituent of China’s energy consumption and the burning of coal releases huge amounts of CO_2_. For this reason, the influence of coal consumption, as a proportion of overall energy use, on CO_2_ emissions cannot be ignored while investigating CO_2_ emissions in China. Es in the study refers to the proportion of coal consumption of overall energy consumption in China (unit: %). Coal consumption data can be found in *China Energy Yearbook*.
(3) Urbanisation level (Ul): this exerts an effect on CO_2_ emissions mainly through changes in land use type and the improvement of Ul, which leads numerous people to change their lifestyles, thus directly improving residential CO_2_ emissions. In this study, the proportion of urban population in the total population is applied to represent Ul (unit: %). The urban population and the total population can be found in local statistical year book.
(4) Trade openness (To): this exerts a significant influence on China’s carbon emissions because China enjoys a trade surplus all year round due to her abundant export volumes. However, a direct negative effect caused by this condition is that it causes domestic CO_2_ emissions and environmental pollution during production while acquiring abundant export exchanges: To is measured by using the ratio of the total export-import volume to GDP (unit: %). The total export-import volume and the province-level GDP can be found in local statistical year book.
(5) Marketisation index (Mi): this refers to a concept used for measuring the capacity to absorb foreign capital inflow by calculating the ratio of foreign direct investment (FDI) to GDP. In this study, a model with Mi was applied to measure the influence of the degree of marketisation on CO_2_ emissions. The ratio of FDI to GDP was used to measure the marketisation index (unit: %). FDI and the province-level GDP can be found in local statistical year book.
(6) Carbon emission *per capita* (Rce): this can be calculated by using the material balance algorithm as recommended by the Intergovernmental Panel on Climate Change (IPCC) (unit: ton/person):
  (5) $$CE=\sum_{i}^{k}EC_{i}*Coe_{i}*\frac{44}{12}$$
  where *CE* is the total CO_2_ emission, and *EC_i_* refers to the consumption of *i*-th energy and *Coe_i_* represents the carbon emissions factor of *i*-th energy. To get carbon emissions per capita (Rce), we divide *CE* by the population. Table 1 is the emission factor of different energy from Compilation of National and Provincial GHG Inventories. Energy consumption data can be found in China Energy Yearbook and province-level population can be found in local statistical year book.

Table 2 shows the descriptive statistical magnitudes of all variables, each of which contains 30 observed values, and 20 observed periods, from 1995 to 2014.

## 4. Empirical Results

### 4.1. Relative Test

González et al. [1] suggested that, before establishing a PSTR model, it is necessary to conduct a heterogeneity test on the model so as to determine the non-linear relationship between explanatory variables and explained variables. Only the mode variables pass through the heterogeneity test, the PSTR model can be established to investigate the non-linear relationship and transfer characteristics of variables.

The homogeneity test is carried out on the PSTR model (1), i.e., we tested the null hypothesis. On the condition of conforming to the null hypothesis *H*_0_, the model (1) is degenerated into a common linear fixed effect model:

(6) $$y_{it}=\mu_{i}+{\beta^{\prime }}_{0}x_{it}+\varepsilon_{it}$$

However, under null hypothesis conditions, the PSTR model contains unidentified nuisance parameters, as first proposed by Davies [25]. Guided by González et al., by substituting the transition function *G* (*q_it_*; *γ, c*) into the first-order Taylor expansion at *γ* = 0*,* the auxiliary equation can be further constructed:

(7) $$y_{it}=\mu_{i}+\beta_{0}^{\prime *}x_{it}+\beta_{1}^{\prime *}x_{it}q_{it}+\cdots +\beta_{m}^{\prime *}x_{it}q_{it}^{m}+\varepsilon_{it}^{*}$$

where, the parameter vector $\beta_{1}^{*},\ldots ,\beta_{m}^{*}$ is the product term of the slope parameter *γ* while $\;\varepsilon_{it}^{*}=\varepsilon_{it}+R_{m}\beta_{1}^{\prime }x_{it}$, in which *R_m_* is the remainder of the Taylor series expansion. Therefore, testing *H*_0_: γ = 0 on model (1) is equivalent to testing $H_{0}^{*}:\;\beta_{1}^{*}=\cdots =\beta_{m}^{*}=0$ on model (5). Thus, by separately estimating the parameters in models (5) and (6), *LM* and *LR* test statistics complying with the *χ*^2^ distribution is constructed based on the residual sums of squares *SSR*_0_ and *SSR*_1_ of the two models to test the null hypothesis *H*_0_:

(8) $$LM=\frac{TN\left(SSR_{0}-SSR_{1}\right)}{SSR_{0}}$$

(9) $$LM_{F}=\frac{(SSR_{0}-SSR_{1})/mk}{SSR_{0}/(TN-N-mk)}$$

(10) $$LR=-2[\log(SSR_{1})-\log(SSR_{0})]$$

where, *SSR*_0_ and *SSR*_1_ refer to the panel residual sums of squares of the null hypothesis (namely, the linear hypothesis) and alternative hypothesis (namely, the hypothesis of the PSTR model), respectively. Under the condition of the null hypothesis, *LM_W_* and *LR* test statistics follow the *χ*^2^ distribution while *LM_F_* statistics conform to an *F* (*mk*,*TN* − *T* − *mk*) distribution.

Table 3 shows that test statistics reject the null hypothesis *H*_0_ at the 1% significance level by taking lnGDPpc as the transition variable, while it also denies the null hypothesis *H*_0_ at the 5% significance level by taking lnEs  and lnUl as transition variables. The rejection of the null hypothesis shows the existence of panel heterogeneity, so the number of transition functions and location parameters needs to be determined by further analysis. Considering the actual conditions, the data used in the study span 20 years (1995 to 2014), so with such a short length time series, it is impractical to have more than two transition functions in this context, or it does not conform to the general laws of economics. Therefore, it is possible to have one or two transition functions. For the number of location parameters, it is possible that *m* = 1 or *m* = 2 in practical situations according to González et al. [1]. Therefore, guided by the methods of Colletaz and Hurlin [26], the most appropriate model is chosen by using different information criteria (Table 4).

### 4.2. Determination of Models

For model 1, by taking lnGDPpc  as the transition variable, it can be seen, through Akaike Information Criterion (AIC) and Bayesian Information Criterion (BIC), that AIC and BIC are minimised at r=2 and  m=1; however, by determining the locations of the transition parameters, it can be seen that location parameter *c* is out with the range of transition variables, so the condition can be excluded. Additionally, r=1,m=2  and  r=2,m=1 have the same AIC and BIC but it is better to choose r=1,m=2  for the sake of model simplification and also to decrease data loss. Namely, the PSTR model established by taking lnGDPpc as the transition variable exhibited a two-regime PSTR relationship and had two transition parameters.

For model 2, taking lnEs as the transition variable, r=1,m=2 and r=2,m=2 can be first excluded because the location parameter *c* is beyond the range of transition variables, which is the same as the analysis taking lnGDPpc as the transition variable. In a similar way, to simplify the model and decrease data loss, r=1,m=1 is chosen in preference, namely, the PSTR model taking lnEs  as the transition variable showed a two-regime PSTR relationship and had one transition parameter.

For model 3, using lnUl as the transition variable, AIC and BIC had their maximum test values at r=2, m=2 and the location parameter was within the range of transition variables. Therefore, a two-regime transition PSTR model was established and each regime contained two location parameters. However, it was worth noting that the result of parameter estimation for this model showed that the four location parameters of the PSTR model established by taking r=2,m=2 satisfied $c_{1}=c_{3},c_{2}=c_{4}$. Namely, $y_{it}=u_{i}+\beta_{0}^{\prime }+2\beta_{1}^{\prime }G\left(Z_{it};\gamma ,c\right)+\varepsilon_{it}$. In fact, it was still the same model at r=1,m=1, so the PSTR model taking lnEs as its transition variable contained a two-regime PSTR relationship and had two location parameters.

By analysing the significance of the aforementioned tests and parameter estimation, the final estimation results of the PSTR model separately taking lnGDPpc, lnEs, and lnUl as transition variables are shown below.

### 4.3. Empirical Results and Discussion

#### 4.3.1. PSTR Model Taking lnGDPpc as the Transition Parameter

By taking GDPpc as the transition variable, the model analyses the influence of changes in GDPpc in different regions of China on *per capita* CO_2_ emissions. It can be seen from Table 5 that whether in low, or high, states, the explanatory variables of the PSTR model all exhibit significance at 1% significance level. The estimation results showed that the influence of $\ln GDPpc_{it}$ on $\ln Rce_{it}$ exhibited the characteristic of having double thresholds (duplication). Firstly, the two thresholds of $\ln GDPpc_{it}$ are $c_{1}=c_{2}=9.7307$, namely, the two thresholds coincided. Moreover, the range of variable values of $\ln GDPpc_{it}$ is [7.5098,12.8188] and the two thresholds are within the acceptable range. Additionally, the transition function $G\left(lnGDPpc_{it};\gamma ,c_{1},c_{2}\right)={\left(1+\exp(-\gamma {\left(\ln GDPpc_{it}-c_{1}\right)}^{2}\right)}^{-1}$ shows the characteristic “first decreasing, then increasing” trend. When $\ln GDPpc_{it}$ was 9.7037, i.e., GDPpc was 16,378 yuan, $\underset{{\mathrm{lnGDPpc}}_{\mathrm{it}}\to \pm \infty }{\lim}G\left({\mathrm{lnGDPpc}}_{it};\gamma ,c_{1},c_{2}\right)=0.5\;$and in this context, the model is in a low regime/state. The model tends to the high regime/state when $\ln GDPpc_{it}$ gradually decreases or increases, namely, $\underset{\ln GDPpc_{it}\to \pm \infty }{\lim}G\left(\ln GDPpc_{it};\gamma ,c_{1},c_{2}\right)=1$. The slope parameter *γ* = 0.6468 is small, which indicates that the model has a slow transition rate between its high- and low-regimes.

Moreover, with the changes in $\ln GDPpc_{it}$, the influence of $\ln GDPpc_{it}\;$on ln*Rce_it_* varied within the range from 0.2304 to 0.6339. This indicated that, as GDPpc gradually increased, the influence coefficient decreased from 0.4322 to 0.2304 although GDPpc showed a pull function effect on *per capita* CO_2_ emissions.

#### 4.3.2. The PSTR Model Taking ln*Es_it_* as the Transition Parameter

By taking Es as the transition variable, the model analyses the non-linear influence of changes in energy consumption structures in different regions of China on *per capita* CO_2_ emissions. Table 5 shows that, regardless of regime/status, GDPpc, Ul, and To in the PSTR model are significant at the 1% level. The estimation showed that the influence of ln*Es_it_* on ln*Rce_it_* exhibited the characteristic of a single threshold. Moreover, the variable values of ln*Es_it_* were within [2.7436, 4.05841] and the thresholds c=4.1241 are within an acceptable range; however, the transition function $G\left(\ln GDPpc_{it};\gamma ,c_{1},c_{2}\right)={\left(1+\exp(-\gamma {\left(\ln GDPpc_{it}-c_{1}\right)}^{2}\right)}^{-1}$ monotonically increases and $\underset{\ln Es_{it}\to \pm \infty }{\lim}G\left(\ln Es_{it};\gamma ,c_{1},c_{2}\right)=0$ when ln*Es_it_* gradually decreases. In this context, the model is in a low regime/status. With the increase of ln*Es_it_*, the model tends to be in its high regime/status and $\underset{\ln Es_{it}\to \pm \infty }{\lim}G\left(\ln Es_{it};\gamma ,c_{1},c_{2}\right)=1$ while $\underset{\ln Es_{it}\to \pm \infty }{\lim}G\left(\ln Es_{it};\gamma ,c_{1},c_{2}\right)=0.5\;$ and $\;\ln Es_{it}=4.1241$. Additionally, the slope parameter *γ* is 1.1481 which indicated that the model exhibited a slow transition between high- and low-regimes, but was slightly quicker compared than that using $\ln Es_{it}$. Upon changes in ln*Es_it_*, the influence of ln*Es_it_* on ln*Rce_it_* varied within the range from −0.6746 to 1.5026. Therefore, it showed that, with the decrease of ln*Es_it_*, the influence of ln*Es_it_* on CO_2_ emissions gradually changed from high- to low-regime status while the degree of this influence gradually changed from 1.5026 to −0.6746. It can be seen that when coal accounts for a large proportion of overall energy consumptions, it exhibited a significant positive effect on CO_2_ emissions, however, as E_S_ gradually decreased, namely, the proportion of coal in the overall energy consumption gradually decreased, not only did the extent of the pull of coal on CO_2_ emissions decrease, but coal exerted a negative influence on CO_2_ emissions after Es exceeded a value such that $\ln Es_{it}=4.1241$. Moreover, with the further decrease of ln*Es_it_*, the negative influence increased because the larger the proportion of coal in overall energy consumptions, the more unreasonable the energy consumption structure of the economy and the greater the CO_2_ emissions produced by coal. However, gradual optimisation of Es not only decreased coal consumption but also improved the efficiency of energy utilisation in the economy and as this promoted the utilisation of new energy sources, CO_2_ emissions were increasingly reduced.

#### 4.3.3. PSTR Model Taking ln*Ul_it_* as the Transition Parameter

The model discusses the non-linear influence of Ul, and the changes therein, in different regions of China on *per capita* CO_2_ emissions by taking Ul as the transition variable. Table 5 shows that, whether in high or low regime status, the explanatory variables of the PSTR model exhibit significance at the 1% significance. The results show that the influence of ln*Ul_it_* on ln*Rce_it_* had the characteristic of double thresholds (duplication). Firstly, the two thresholds of ln*Ul_it_* can be expressed as $c_{1}=c_{2}=3.7997$, namely, the two thresholds coincided. Moreover, the variable values of ln*Ul_it_* were within [2.8443, 4.4953] and the two thresholds were both within their acceptable range. However, the transition function $G\left(lnUl_{it};\gamma ,c_{1},c_{2}\right)={\left(1+\exp(-\gamma {\left(\ln Ul_{it}-c_{1}\right)}^{2}\right)}^{-1}$ decreased first and then increased and when ln*Ul_it_ = 3.7997*, there existed $\underset{\ln Ul_{it}\to \pm \infty }{\lim}G\left(\ln Ul_{it};\gamma ,c_{1},c_{2}\right)=0.5$. In this context, the model is in a low regime status. When ln*Ul_it_* gradually decreased, or increased, the model tended to its high regime status, namely, $\underset{\ln Ul_{it}\to \pm \infty }{\lim}G\left(\ln Ul_{it};\gamma ,c_{1},c_{2}\right)=1$. The slope parameter γ took the large value of 10.8779, which indicated that the model had a rapid transition rate between its high- and low-regimes. With changes in ln*Ul_it_*, the influence of ln*Ul_it_* on ln*Rcel_it_* changed within the range from 0.1563 to 0.4374, which indicated that, with gradual improvements in Ul, the influence coefficient decreased from 0.4374 to 0.1563 although urbanisation generally exerts a pull effect on *per capita* CO_2_ emissions, it shows that the pull effect of ln*Ul_it_* on ln*Rce_it_* decreased. The primary reason for this was that, in the early stage of urbanisation, various advantages of cities including the cluster effect had not yet appeared and urban expansion, in its early stage, requires that more infrastructure be built; however, when urbanisation reaches a certain level, the scale of urbanisation, its structure, and infrastructure are relatively mature while the equivalent scale expansion of urbanisation requires little boundary investment. Additionally, there is a synergistic effect between cities and towns and the occurrence of city groups further reduces unit carbon emissions levels.

## 5. Conclusions

There has been much research into the relationship between CO_2_ emissions and economic growth, which remains a problem worthy of discussion. Many previous studies do not take account the non-linearity of the relationship. This study investigated a possible non-linear relationship between economic growth and CO_2_ emissions in China fitted using the PSTR model. Additionally, considering possible missing variables, multiple variables were introduced including Es, Ul, To, and Mi while three variables were used as transition variables to establish the models.

The main results were as follows: for the PSTR model taking GDPpc, Es, and Ul as thresholds, the influences of the three factors on *per capita* CO_2_ emissions exhibited non-linear panel smooth transition characteristics. The transition rate of the influence of Ul on *per capita* CO_2_ emissions was significantly higher than that of GDPpc and Es. For the PSTR model using GDPpc as the threshold, the influence of GDPpc on *per capita* CO_2_ emissions showed the characteristic of coincident double thresholds. Moreover, GDPpc exerted a pull effect on *per capita* CO_2_ emissions as it gradually increased, but that influence coefficient gradually decreased from 0.4322 to 0.2304, which indicated that the pull effect of GDPpc on *per capita* CO_2_ emissions gradually declined. It could be seen from the empirical results that, for the relationship between economic development and carbon emissions, government should pay attention to the various environmental pressures caused by Ul such as the growth of carbon emissions generated while improving Ul. With the development of the economy and GDPpc, the pull effect of GDPpc on *per capita* CO_2_ emissions gradually declined. However, it was necessary to concentrate on the absolute amount of carbon emissions and it was unreasonable to follow the path of developing the economy first and then dealing with the pollution as an afterthought. The results of empirical analysis show that with the development of economy, CO_2_ emission per capita is growing in absolute amount, but the pull effect is declining. Based on that, the Chinese government could maintain its economic growth goal while cutting carbon emissions through changing energy consumption structure, i.e., using less coal and more clean energy. Chinese government plays a central role in promoting urbanization, when the process reaches a certain level, carbon emissions began to decline due to a synergistic effect between cities and towns. The government could formulate an urbanization plan which is environment-friendly to cut carbon emissions.

## Figures and Tables

**Table 1:** Carbon emissions factor.

Energy	Coal	Coke	Crude Oil	Gasoline	Kerosene	Diesel Oil	Fuel Oil	Nature Gas
carbon emissions factor	1.9003	2.8604	3.0202	2.9251	3.0179	3.0959	3.1705	2.1622

Note: The unit of nature gas carbon emission factor is kgCO_2_/m^3^, others is kgCO_2_/kg.

**Table 2:** Statistical magnitudes of all variables.

Variables	Rce	GDPpc	Es	Ul	To	Mi
Observation	600	600	600	600	600	600
Mean	6.64	16,247.37	69.76	44.85	30.92	7.03
Standard deviation	5.180	27,093	16.652	16.634	39.861	7.9899
Maximum	31.20	369,120.78	97.92	89.6	205.12	49.51
Minimum	0.737	1826	15.543	17.19	0.336	0.067

**Table 3:** Non-linear testing of models.

Model	1	2	3
threshold variables	lnGDPpc	lnEs	lnUl
$H_{0}:r=0\;\mathrm{vs}.\;H_{1}:r=1$			
*LM*	150.772	153.199	83.540
	(0.000)	(0.000)	(0.000)
*LM_F_*	18.795	38.745	18.278
	(0.000)	(0.000)	(0.000)
*LR*	173.639	176.889	89.959
	(0.000)	(0.000)	(0.000)

Note: the values in parentheses refer to significance level *p*.

**Table 4:** Determination of model parameters.

Model	1				2				3			
Threshold variables	lnGDPpc				lnEs				lnUl			
(*r*, *m*)	(1, 1)	(1, 2)	(2, 1)	(2, 2)	(1, 1)	(1, 2)	(2, 1)	(2, 2)	(1, 1)	(1, 2)	(2, 1)	(2, 2)
AIC	−3.54	−3.57	−3.60	−3.57	−3.60	−3.59	−3.60	−3.59	−3.54	−3.61	−3.64	−3.61
BIC	−3.45	−3.48	−3.46	−3.48	−3.51	−3.50	−3.51	−3.50	−3.45	−3.51	−3.50	−3.51
Is the location parameter out the of region?	Yes	No	Yes	No	Yes	No	No	Yes	No	No	Yes	No
Final model (*r*, *m*)	(1, 2)				(1, 1)				(1, 2)			

Note: *r* and *m* represent the numbers of transition functions and location parameters, respectively.

**Table 5:** Estimation results of the PSTR model.

Model	1		2		3	
Threshold Variables	lnGDPpc		lnEs		lnUl	
	β	β ^(1)^	β	β ^(1)^	β	β ^(1)^
lnGDPpc	0.6339 ***	−0.4035 ***	−0.6746 ***	2.1772 ***	0.7185 ***	−0.5622 ***
	(6.8580)	(−3.4128)	(−5.7824)	(9.1263)	(7.8991)	(−4.3070)
lnEs	−0.1592 ***	0.1921 **	−0.2218	0.3254	−0.1660 ***	0.1779 **
	(−3.0084)	(2.4590)	(−1.4833)	(1.1975)	(−3.1589)	(2.4991)
lnUl	−0.6490 ***	1.3625 ***	−3.5520 ***	1.7178 ***	−0.6829 ***	1.3278 ***
	(−4.0785)	(10.8281)	(−3.0020)	(5.8940)	(−4.6742)	(9.5960)
$\ln T_{o}$	1.4856 ***	−0.9914 **	2.9676 ***	−4.4498 ***	1.4559 ***	−0.3981
	(5.4447)	(−2.7422)	(3.9627)	(−3.3184)	(4.9173)	(−1.0857)
$\ln M_{i}$	−0.2777 ***	0.3182 ***	−0.2874	0.5500	−0.2496 ***	0.2958 ***
	(−4.2554)	(3.0523)	(−1.3856)	(1.4246)	(−4.4999)	(3.8394)
γ	0.6468		1.1481		10.8779	
*c*	$c_{1}=c_{2}=9.7307$		c=4.1241		$c_{1}=c_{2}=3.7997$	
*RSS*	15.718		15.408		15.165	

Note: The values in parentheses refer to values of *t*; ***, **, and separately refer to the presence of significance at 1%, 5%; β ^(1)^ separately represents the estimation coefficients of interaction between various explanatory variables and the first transition function; transition functions are all expressed in the form of logistic functions.
